# γ-Secretase Dependent Production of Intracellular Domains Is Reduced in Adult Compared to Embryonic Rat Brain Membranes

**DOI:** 10.1371/journal.pone.0009772

**Published:** 2010-03-19

**Authors:** Jenny Frånberg, Helena Karlström, Bengt Winblad, Lars O. Tjernberg, Susanne Frykman

**Affiliations:** Karolinska Institutet Alzheimer's Disease Research Center (KI-ADRC), Department of Neurobiology, Care Sciences and Society (NVS), Karolinska Institutet, Huddinge, Sweden; Brunel University, United Kingdom

## Abstract

**Background:**

γ-Secretase is an intramembrane aspartyl protease whose cleavage of the amyloid precursor protein (APP) generates the amyloid β-peptide (Aβ) and the APP intracellular domain. Aβ is widely believed to have a causative role in Alzheimer's disease pathogenesis, and therefore modulation of γ-secretase activity has become a therapeutic goal. Besides APP, more than 50 substrates of γ-secretase with different cellular functions during embryogenesis as well as adulthood have been revealed. Prior to γ-secretase cleavage, substrates are ectodomain shedded, producing membrane bound C-terminal fragments (CTFs).

**Principal Findings:**

Here, we investigated γ-secretase cleavage of five substrates; APP, Notch1, N-cadherin, ephrinB and p75 neurotrophin receptor (p75-NTR) in membranes isolated from embryonic, young or old adult rat brain by analyzing the release of the corresponding intracellular domains (ICDs) or Aβ40 by western blot analysis and ELISA respectively. The highest levels of all ICDs and Aβ were produced by embryonic membranes. In adult rat brain only cleavage of APP and Notch1 could be detected and the Aβ40 and ICD production from these substrates was similar in young and old adult rat brain. The CTF levels of Notch1, N-cadherin, ephrinB and p75-NTR were also clearly decreased in the adult brain compared to embryonic brain, whereas the APP CTF levels were only slightly decreased.

**Conclusions:**

In summary our data suggests that γ-secretase dependent ICD production is down-regulated in the adult brain compared to embryonic brain. In addition, the present approach may be useful for evaluating the specificity of γ-secretase inhibitors.

## Introduction

γ-Secretase is an intramembrane aspartyl protease which plays a pivotal role in Alzheimer's disease since it mediates the release of the amyloid β-peptide (Aβ) from the amyloid precursor protein (APP) [1]. Fibrils of polymerized Aβ are deposited extracellularly as senile plaques, one of the hallmarks of AD affected brains [2]. In addition, the pre-fibrillar oligomeric Aβ is believed to contribute to the pathogenic mechanisms that lead to synaptic dysfunction and neuronal loss in AD [3], [4]. Prior to γ-secretase cleavage, the ectodomain of APP is shedded by β-secretase or α-secretase [5], [6], generating membrane bound C-terminal fragments (CTFs) which are further processed by γ-secretase. The sequential processing of APP by β- and γ-secretase results in Aβ peptides of various lengths, of which the 40 or 42 residue peptides are the most abundant [7]. The latter aggregates more easily and is the species implicated early in plaque formation. In addition to the Aβ-generating cleavage site in the middle of the membrane, γ-secretase cleaves APP CTFs close to the membrane-cytoplasm boundary (ε-site cleavage), generating the APP intracellular domain (AICD) [8], [9].

γ-Secretase is composed of at least four subunits, presenilin (PS), nicastrin, anterior pharynx defective-1 (Aph-1) and presenilin enhancer-2 (Pen-2) [10], [11], possibly in a 1∶1∶1∶1 stoichiometry [12]. PS is endoproteolytically cleaved into N- and C-terminal fragments (PS-NTF and PS-CTF) [13] during maturation to an active complex. There exist two homologues of PS, presenilin-1 (PS1) and presenilin-2 (PS2) and two homologues of Aph-1 in humans, Aph-1a and Aph-1b [14]. In addition, there are two splice variants of Aph-1a. PS1 and PS2 do not coexist in the same γ-secretase complex and neither do the different Aph-1 isoforms. Thus, at least six distinct complexes with different subunit composition might form [14]–[17].

In addition to APP, γ-secretase cleaves more than 50 other substrates [18] in a similar manner, among which the Notch receptor is the most characterized. Upon maturation, the Notch receptor is cleaved by furin-like convertases, at the S1 site, forming a heterodimer that is targeted to the plasma membrane. Activation of Notch, by ligand binding, induces cleavage by ADAM metalloproteases at the extracellular S2 site resulting in a membrane-anchored truncated form of Notch, which is processed by γ-secretase (sites S3 and S4) [19]. The ε-cleavage in APP corresponds to the S3 cleavage of Notch which results in the liberation of the Notch intracellular domain (NICD) which enters the nucleus and regulates transcription [20]–[22]. The Notch pathway controls a broad range of events during embryonic and adult development such as cell fate decisions and proliferation [23], [24]. Notch1 deficiency is an embryonic-lethal phenotype associated with severe developmental deficits, including disturbed somitogenesis [25], [26]. N-cadherin, ephrinB and p75 neurotrophin receptor (p75-NTR) are three other important γ-secretase substrates [27]–[31]. N-cadherin is a cell adhesion molecule which regulates synaptic contact, synaptogenesis, long-term potentiation and morphology of the dendritic spines. Interaction of ephrinB with ephrinB receptors is essential for the development of the vascular system and the nervous system, and in the adult for the regulation of synaptic plasticity. p75-NTR is involved in neuronal death during development and following injury and also in neuronal differentiation and axon elongation. In analogy with NICD, it is possible that γ-secretase released intracellular domains (ICDs) from other substrates also play a role in signal transduction/transcriptional activation [32]. For example it has been reported that AICD can regulate the transcription of the tumor suppressor p53 [33]. In addition, N-cadherin ICD has been shown to bind and promote degradation of the transcription factor CREB binding protein [29]. γ-Secretase mediated intramembrane proteolysis may also be involved in non-nuclear signaling functions such as formation/disassembly of receptor complexes (p75-NTR) or src activation (ephrinB) [27], [30]. Another suggested role of γ-secretase is as the proteasome of the membrane, removing the substrates transmembrane domains [34]. γ-Secretase cleaves with loose sequence specificity and no consensus sequence within the substrates has been identified. The substrates are with few exceptions type I transmembrane proteins that have undergone ectodomain shedding. However, the subunit composition of γ-secretase can regulate activity, cleavage site and substrate specificity. The PS1 and PS2 complexes show differences in Aβ production and sensitivity to certain γ-secretase inhibitors [35] and PS1 deficiency results in a more severe phenotype than PS2 deficiency [36]–[38]. Further, Aph-1a seems to be the major isoform required for γ-secretase activity during mouse embryonic development [39] while Aph-1b is suggested to be involved in γ-secretase cleavage of neuregulin-1 [40]. Hence, different γsecretase complexes seem to have different properties and physiological functions. Interestingly, γ-secretase inhibitor binding density has been shown to be higher in postnatal rat brains than in adult [41] suggesting that γ-secretase could be developmentally regulated as well. Furthermore, γ-secretase substrate selectivity could be regulated spatially through the association of γ-secretase and its substrates in lipid rafts [42]–[44]. During development, γ-secretase has been suggested to reside in non-raft membranes together with several substrates but being translocated to lipid rafts in adult brain [42].

Since γ-secretase executes the final step in the APP processing, thereby generating the pathogenic Aβ peptides, modulation of its activity for therapeutic purposes is extensively investigated. The multitude of substrates makes this task difficult due to the risk of side effects. Therefore, relevant assays for studying the cleavage of different γ-secretase substrates are important. Most assays that exist today are based on cell lines often over-expressing the substrate. To our knowledge there are no extensive studies in brain tissue on γ-secretase cleavage of endogenous substrates other than APP, and it is not known whether this cleavage is developmentally regulated. Therefore, we have here studied the cleavage of five γ-secretase substrates important for neuronal function; APP, Notch1, N-cadherin, ephrinB and p75-NTR. The substrate cleavage was examined in membrane preparations from embryonic (E17), 2–3 month-old and 16–18 month-old rat brain. γ-Secretase cleavage of all of the examined substrates was detected in embryonic rat brain while only processing of APP and Notch1 was detected in the adult brain and to a much lower extent.

Here we conclude that γ-secretase dependent ICD production is decreased in adult rat brain membranes compared to embryonic.

## Results

### γ-Secretase cleavage of endogenous Notch1 in membrane preparations from rat brain

**Figure 1:**
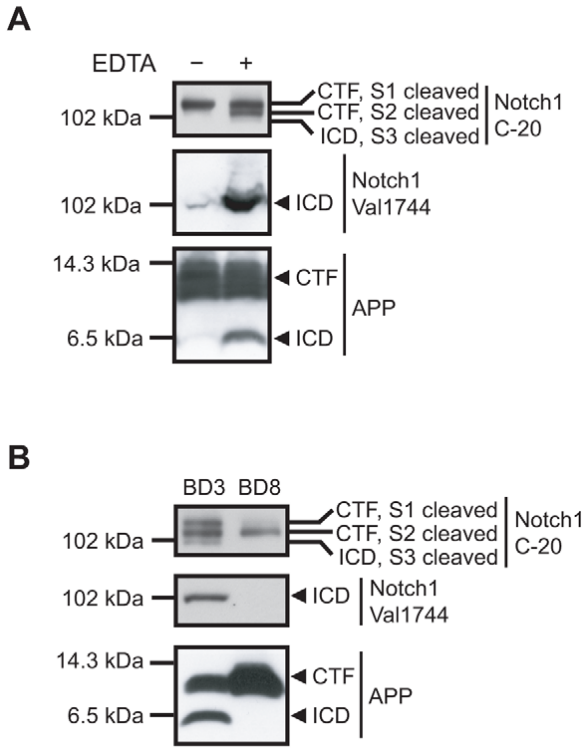
γ-Secretase cleavage of endogenous Notch1 and APP in adult rat brain or BD3 cells. (A) Adult rat brains (3 month-old) were homogenized and membranes were isolated by sequential centrifugations at 1000×g and 100 000×g. Membranes were incubated for 16 h at 37°C with or without 5 mM EDTA. The samples were subjected to SDS-PAGE and western blotting using the antibodies Notch1 C-20, directed to the C-terminal of Notch1, Val1744, only recognizing the free N-terminal of NICD, or C1/6.1, directed to the C-terminal of APP. (B) BD3 and BD8 cells were harvested in PBS and membranes were prepared and incubated in buffer H containing 5 mM EDTA for 16 h at 37°C. The samples were analyzed as described above.

**Figure 2:**
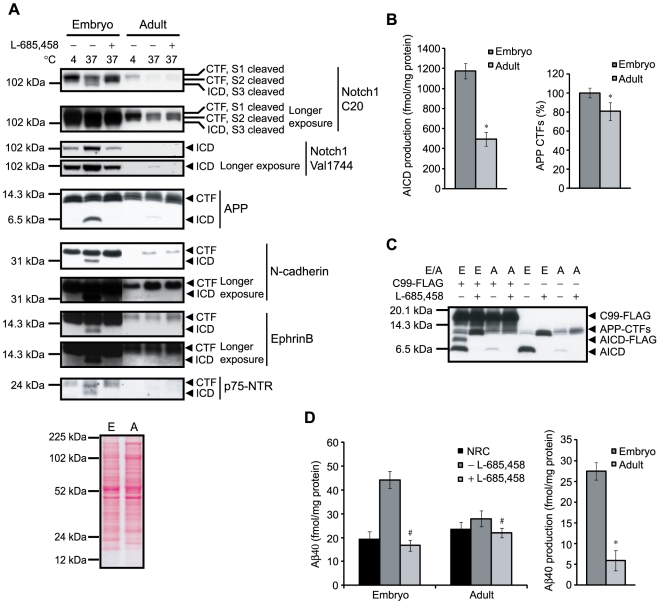
Comparison of γ-secretase dependent ICD and Aβ40 production in embryonic and adult rat brain. (A) Membranes were prepared from embryonic (day 17) and adult rat brains (3 month-old) and equal amounts of protein from the two preparations were incubated at 4°C or at 37°C in the absence or presence of the γ-secretase inhibitor L-685,458. The samples were separated by SDS-PAGE and analyzed by western blot for ICDs from APP, Notch1, N-cadherin, ephrinB and p75-NTR. The different ICDs as well as CTFs were detected by C-terminal antibodies for the different substrates. NICD was detected also by an antibody, Val1744, only recognizing the neoepitope generated following cleavage at the S3 site. The embryonic and adult lanes are in all cases from the same western blot. To confirm that the amount of protein loaded onto the gel and transferred to membrane was similar in embryonic (E) and adult (A) membrane preparations Ponceau S staining was used (lower panel). (B) Quantification of AICD production and APP CTFs in embryonic and adult rat brain membranes. The AICD levels in the samples incubated at 37°C (left panel) and the APP CTF levels in the non-incubated sample (4°C) were quantified using two standard points of synthetic AICD peptide. Data are presented as mean values ± SD (n = 4). *, p<0.05 adult vs embryo. (C) Embryonic (E) and adult (A) rat brain membranes were assayed for γ-secretase activity as above in the presence or absence of C99-FLAG. Generation of endogenous AICD and AICD-FLAG was detected by the antibody C1/6.1, directed to the C-terminal of APP. (D) The Aβ40 levels were measured by sandwich ELISA in embryonic and adult rat brain membrane preparations containing equal amounts of protein. The membranes were incubated for 8 h at 37°C in the absence or presence of the γ-secretase inhibitor L-685,458. The no reaction control (NRC) samples were excluded from incubation and instead treated directly with RIPA buffer to inhibit further production of Aβ. Production (right panel) was determined by subtracting Aβ40 values obtained in the inhibited samples (+ L-685,458) from samples without inhibitor (− L-685,458). Data are presented as mean value ± SD (n = 4). *, p<0.05 adult vs embryo; #, p<0.05+ L-685,458 vs−L-685,458 within the same age group.

To study the cleavage of γ-secretase substrates other than APP, we first optimized the buffer conditions for the activity assay. The metal chelator EDTA has been shown to efficiently block the degradation of AICD *in vitro* in membranes prepared from HEK293 cells [45], [46]. In addition, EDTA has been shown to induce shedding of the Notch receptor (S2 cleavage) and thereby signaling (S3 cleavage) in a ligand-mimicking manner in cell systems [47], [48]. Here, we examined the effect of EDTA on AICD and NICD in rat brain membranes. Membrane fractions from adult rat brains were isolated by sequential centrifugation steps. Nuclei and non-homogenized cells were removed by centrifugation at 1000×g and membranes were collected by centrifugation at 100,000×g. The membranes were incubated at 37°C for 16 h in Hepes buffer in the absence or presence of 5 mM EDTA and the production of NICD and AICD, as well as the substrate levels, were estimated by western blot analysis. EDTA increased the levels of both AICD and NICD (Figure 1A), but in sodium citrate buffer at pH 6.4 (previously used for studying γ-secretase activity [29], [46]) this effect was less pronounced (data not shown). Even in the EDTA treated samples, NICD was barely detected, using an antibody directed to the C-terminal of Notch1, C-20. However, using a NICD specific antibody, Val1744, NICD was readily detected. As previously reported, we noted that EDTA was necessary for the S2 cleavage of Notch1 to occur (Figure 1A), and 5 mM EDTA was added in the subsequent activity experiments. The EDTA induced production of the S2 cleaved Notch1 fragment was not inhibited by the γ-secretase inhibitor L-685,458 (Figure 2A).

We also wanted to investigate whether cleavage of Notch1 and APP could be detected in a commonly used cell-line in the γ-secretase field, the BD3 cells, lacking PS1 at one allele and both alleles of PS2. As a negative control, BD8 cells which lack both PS1 and PS2 were included. Indeed, we could detect cleavage of endogenous APP and Notch1 in BD3 but not in BD8 cells (Figure 1B). This cleavage was γ-secretase dependent since it was inhibited by L-685,458 (data not shown). The level of S2 cleaved Notch1 was similar in membranes from BD3 and BD8 cells. However, the level of S1 cleaved Notch1 was lower in the BD8 cells compared to BD3 cells. This could possibly be explained by disruption of a positive feedback-loop in which Notch signalling upregulates Notch mRNA expression. Indeed, activation of the *Caenorhabditis elegans* homologue to Notch, Lin-12, has previously been shown to be important for maintaining Lin-12 mRNA expression [49]. As expected for the BD8 cells, the relative level of the S2 fragment (S2/S1) is increased since the γ-secretase dependent processing of this fragment is abolished in these cells.

### Generation of ICDs from endogenous γ-secretase substrates in embryonic and adult rat brain membranes

Next, we studied γ-secretase cleavage of different endogenous substrates at different stages of development. We chose five different neuronal substrates with important functions in the developing and adult nervous system and studied the cleavage of these in membrane preparations from E17 and 3 month-old rats. The membranes were incubated at 37°C for 16 h in the absence or presence of the γ-secretase inhibitor L-685,458. The production of ICDs from APP, Notch1, N-cadherin, ephrinB and p75-NTR was examined using western blot analysis. Interestingly, we could detect cleavage of N-cadherin, ephrinB and p75-NTR in the embryonic rat brain membranes but not in the adult rat brain (Figure 2A). However, substrate (CTF) levels per µg of protein were also higher in the embryonic membrane preparation compared to adult. APP and Notch1 processing was observed in both embryonic and adult rat brain membranes but to a higher extent in the embryonic rat brain membranes. The substrate levels of Notch1 (S1 and S2 cleaved) were also higher in embryonic rat brain membranes (Figure 2A). For APP, there was an approximately three-fold decrease between embryo and adult AICD production although there was only a 20% decrease in CTF levels (Figure 2B). Ponceau S staining confirmed that the total amount of protein loaded onto the gel was similar in embryonic and adult membrane preparations (Figure 2A, lower panel). To estimate the γ-secretase activity, independent of substrate levels, we added an exogenous APP substrate, C99-FLAG, to the reaction. The production of AICD-FLAG was detected by western blot and was found to be higher in embryonic rat brain than in adult, which indicates that the total γ-secretase activity is higher in embryonic rat brain (Figure 2C). Thus, for several substrates, the decreased production of ICDs in adult brain membranes is probably mainly due to lower substrate levels but for APP this is not the full explanation.

The generation of all of the different ICDs was inhibited by L-685,458, showing that the processing is indeed γ-secretase dependent. There was no significant difference in NICD levels between the samples incubated at 37°C and NRC (Figure 2A), indicating that there was no degradation of NICD during incubation. Furthermore, when L685,458 was added to the reaction after 16 h and the sample were incubated for an additional 16 h, there was no change in the AICD levels during the latter period in either the embryonic or in the adult rat brain membranes. The addition of 10 µM of the protease inhibitors antipain, bestatin and leupeptin and 5 mM EGTA to buffer H (containing Complete™ protease inhibitor cocktail from Roche and 5 mM EDTA) did not alter the levels of AICD in the adult brain. In addition, the ICDs of N-cadherin, p75-NTR and ephrinB were still not detectable (data not shown). These results suggest that the decreased ICD levels in the adult were not due to increased ICD degradation.

### Aβ40 production in embryonic and adult rat brain membranes

Since there was a marked difference in the production of AICD between embryonic and adult rat brain we continued by examining whether the production of Aβ also differed. Membranes from embryonic and adult rat brain were incubated for 16 h at 37°C in the absence or presence of L-685,458 and the samples were analyzed by sandwich ELISA. Unfortunately we could only quantify the Aβ40 and not the Aβ42 levels in this system. In order to measure the starting levels of Aβ40, RIPA buffer was immediately added to NRC samples which were kept at 4°C prior to analysis. Contrary to AICD and NICD, we noticed a decrease in Aβ levels in samples incubated with L685,458 at 37°C for 16 h compared to NRC, indicating some degradation of Aβ40 during incubation. This was also the case for added synthetic Aβ1-40 (data not shown). Since neprilysin and insulin degrading enzyme (IDE) are two major Aβ degrading enzymes [50], [51], we added inhibitors to these proteases (thiorphan and 1,10-phenanthroline respectively) to the samples to prevent degradation. We also decreased the incubation period to 8 h. In this case there was no significant degradation of Aβ (Figure 2D, left panel, + L685,458 compared to NRC). Using these conditions we compared the Aβ40 production in embryonic and adult rat brain membranes and found it to be four to five times higher in embryonic rat brain membranes compared to adult (Figure 2D).

### Levels of γ-secretase components and active γ-secretase in embryonic and adult rat brain

**Figure 3:**
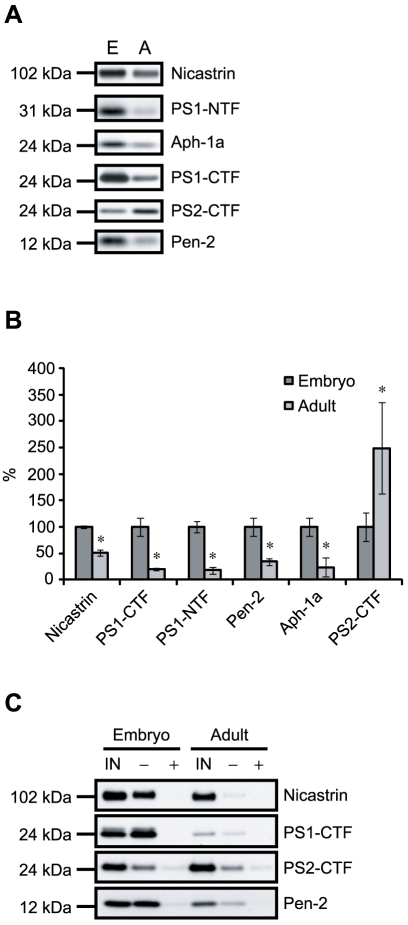
Levels of γ-secretase components and active complexes in embryonic and adult rat brain. (A) The levels of γ-secretase components in equal amounts of protein from embryonic (E) and adult (A) membrane preparations were analyzed by western blot using antibodies directed to the γ-secretase complex components. (B) Quantification of γ-secretase components in embryonic and adult membrane preparations from western blot. Equal amounts of protein from four different embryonic and adult membrane preparations were loaded together with three different dilutions of one of the membrane samples. The intensities from the diluted samples were plotted as a standard curve to which the other membrane preparations were correlated. Data are presented as mean values ± SD (n = 4). *, p<0.05 adult vs embryo (C) Active γ-secretase was captured from embryonic and adult rat brain membranes with 200 nM of a biotinylated γ-secretase inhibitor in the presence or absence of 10 µM L-685,458 followed by the addition of streptavidin beads. Bound complexes and 10% of the input (IN) were separated on SDS-PAGE and detected by western blot using antibodies directed to the γ-secretase components.

To investigate whether the decreased γ-secretase activity could be due to lower levels of γ-secretase, we compared the levels of γ-secretase components in membranes prepared from embryonic and adult rat brain. Most of the γ-secretase components were present to a higher degree in embryonic rat brain membranes (Figure 3A and B). Interestingly however, the PS2-CTF level was higher in the adult rat brain membranes. Further, we investigated whether the γ-secretase components were degraded during the incubation in the activity assay but we found no significant difference in component levels between samples incubated at 37°C for 16 h and non-incubated samples (data not shown).

We also investigated whether the total amount of γ-secretase components reflected their incorporation into active γ-secretase complexes. For this purpose we used affinity capture where the membranes from embryonic and adult rat brain were incubated in the presence of a γ-secretase inhibitor with a cleavable biotin group (GCB) followed by incubation with streptavidin beads to capture the γ-secretase complexes. GCB is based on L685,458, which efficiently inhibits processing of all the substrates tested (Fig. 2A). In addition, GCB itself efficiently blocked Aβ production with a similar IC50 as L685,458 [52]. Thus, GCB is efficient in capturing active γ-secretase complexes. As a negative control L-685,458 was added prior to the biotinylated inhibitor. The bound γ-secretase was eluted from the beads and analyzed by SDS-PAGE and western blotting. Indeed, the higher levels of γ-secretase components found in embryonic rat brain also correlated with higher levels of captured active γ-secretase complex and the differences were even more pronounced in this case (Figure 3C). The levels of captured PS2-CTF were similar in embryonic and adult rat brain (Figure 3C).

### γ-Secretase cleavage of Notch1 and APP in 2–3 month-old and 16–18 month-old rat brain membranes

**Figure 4:**
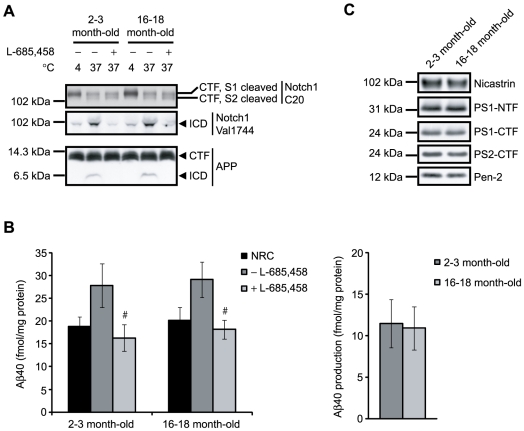
NICD, AICD and Aβ40 production and γ-secretase components in 2–3 and 16–18 month-old rat brains. (A) Membranes were isolated from 2–3 (young) and 16–18 month-old (old) rat brains, incubated for 16 h at 4°C or at 37°C in the absence or presence of the γ-secretase inhibitor, L-685,458, and subjected to SDS-PAGE and western blot analysis. NICD and AICD production was detected using the antibodies, Val1744 and C1/6.1 respectively. The Notch1 CTFs (S1 and S2 cleaved) were detected by the antibody Notch1 C-20. (B) Equal amounts of protein from membranes from 2–3 and 16–18 month-old rat brain were subjected to western blot analysis of the γ-secretase components nicastrin, PS1, PS2 and Pen-2. (C) Membrane preparations were either incubated for 8 h at 37°C with or without the γ-secretase inhibitor L-685,458 or directly treated with RIPA buffer (NRC, no reaction control). Levels of Aβ40 were measured by sandwich ELISA. Production (right panel) was determined by subtracting Aβ40 values obtained in the inhibited samples (+ L-685,458) from samples without inhibitor (− L-685,458). Data are presented as mean value ± SD (n = 4). #, p<0.05+ L-685,458 vs−L-685,458 within the same age group.

To further investigate the effect of age on γ-secretase cleavage of the different substrates we compared γ-secretase activity in 2–3 and 16–18 month-old rat brain. In this comparison we used frozen brains purchased from Rockland Immunochemicals Inc. Membranes were prepared and the activity assay was performed as described above. The generation of AICD, NICD and Aβ40, as well as the levels of APP CTFs and S2 cleaved Notch1, were similar in 2–3 month-old and 16–18 month-old rat brains (Figure 4A and B). The same conditions were used for the young-old comparison as for the embryo-adult comparison. The total γ-secretase activity, assessed by the addition of C99-FLAG, was also similar between the age groups (data not shown). Furthermore, the γ-secretase component levels did not differ substantially between the two age groups (Figure 4C). No ICD production from ephrinB, N-cadherin or p75-NTR was observed in membranes from 2–3 month-old or 16–18 month-old rats (data not shown).

## Discussion

γ-Secretase is responsible for intramembrane cleavage of various type I transmembrane proteins following shedding of their ectodomains. The functional diversity of the substrates, including cell fate determination, cell-cell adhesion, neuritogenesis and synapse formation, emphasizes the importance of γ-secretase during development as well as in adult life. Previously, γ-secretase cleavage has mostly been studied in cell lines where in many cases the substrates have been over-expressed. In this report we demonstrate γ-secretase cleavage of five substrates; APP, Notch1, N-cadherin, ephrinB and p75-NTR, in embryonic rat brain membranes but only cleavage of APP and Notch1 in adult rat brain membranes. The γ-secretase processing of the substrates was monitored by comparing the levels of formed ICDs from the different substrates using western blot analysis. The finding that NICD production can be observed from endogenously expressed Notch1 in rat brain membranes could be useful for developing relevant screening assays for selective γ-secretase inhibitors/modulators. We also examined NICD production in BD3 (PS1^+/−^; PS2^−/−^) and BD8 cells (PS1^−/−^; PS2^−/−^) without overexpression of Notch. No NICD was produced in BD8 cells but by introducing one allele of PS1 as in the BD3 cells NICD production was observed. Thus, the influence of wild type, mutated or truncated PS1 or PS2 on substrate specificity (APP versus Notch processing), could be examined by introducing these PS variants in BD8 cells.

Further, we compared γ-secretase cleavage of endogenous APP, Notch1, N-cadherin, ephrinB, and p75-NTR in membranes prepared from embryonic and adult rat brain and found that cleavage of all of these substrates, i.e. ICD production, was higher in embryonic brain. The production of Aβ from APP was also higher in embryonic brain. For Notch1, the high level of processing in the embryonic brain is in line with the critical role of NICD in cell fate determination during embryogenesis. The other substrates also have important functions during development but it is not fully known to which extent these functions are γ-secretase dependent. The higher ICD production in embryonic rat brain membranes could for Notch1, N-cadherin, ephrinB and p75-NTR be explained by the higher CTF levels of the different substrates. For APP, however, this is not the case since APP CTFs were only slightly altered between embryonic and adult brain. This is in accordance with our previous studies showing that APP CTFs are present even after long incubation times and that γ-secretase appears to be the rate-limiting step in endogenous APP processing [53]. One explanation for the reduced APP cleavage in adult brain despite the presence of the substrate could be that γ-secretase and APP are spatially separated in the membranes. Indeed, we have earlier demonstrated that whereas γ-secretase is located to lipid rafts in adult rat brain membranes, full length APP and APP CTFs are located outside the rafts [43]. This is in contrast to Vetrivel *et al.* who showed that the APP CTFs were localized in lipid rafts [42]. The discrepancy could be due to the different detergents used for lipid raft preparation in the two studies. The γ-secretase processing of an exogenous APP based substrate, C99-FLAG was also decreased in the adult brain membranes showing that substrate concentration per se is not a limiting factor. The higher γ-secretase activity in membranes from embryonic rat brain is in line with the reported higher levels of transition state γ-secretase inhibitor binding in postnatal rat brain compared to adult [41]. The finding that, of the substrates examined, only γ-secretase cleavage of APP and Notch1 was detected in the adult is also in line with the side-effects observed following γ-secretase inhibitor administration. These side-effects, including gastrointestinal toxicity and interference with B- and T-lymphocyte maturation, seem mainly to be due to inhibition of Notch processing and signaling [54]–[56].

Aβ has been shown to be degraded by several proteases [57], including IDE [50] and neprilysin [51]. Apart from Aβ, IDE has also been shown to degrade AICD [46]. To rule out the possibility that the decreased ICD and Aβ levels in adult brain membranes could, at least partly, be due to increased degradation rather than decreased production we compared the level of produced AICD after 16 h with that in samples further incubated in the presence of γ-secretase inhibitor for 16 h. No degradation of AICD was observed in either embryonic or adult membrane preparations. For NICD, we did not find any differences between samples that were incubated at 37°C in the presence of a γ-secretase inhibitor and the samples that were not incubated at all, indicating that degradation in this case is limited. For Aβ, on the other hand, we had to decrease the incubation time from 16 to 8 h and add the neprilysin inhibitor thiorphan and the metalloprotease inhibitor 1,10-phenanthroline to abolish the degradation. These precautions increased the Aβ levels in the embryonic brain more than in the adult brain (data not shown), indicating that the degradation was higher in embryonic brain. Therefore, the decreased levels in the adult were not due to degradation. We also investigated whether the γ-secretase components were degraded, by comparing samples incubated at 4°C and 37°C, and found this not to be the case. Thus, the lack of degradation makes our *in vitro* system suitable for studying γ-secretase dependent production of different ICDs and Aβ. However, *in vivo* the situation is more complex, and the levels of the ICDs and Aβ in the brain could be affected by proteases that are not present in our assay where the cytoplasm has been removed.

One explanation for the decreased γ-secretase activity in adult could be that the levels of the γ-secretase components are decreased. Indeed, we found decreased levels of all γ-secretase components except PS2-CTF in the adult brain. Interestingly, the levels of PS2 were increased. This might be of importance since an increase in PS2 containing γ-secretase complexes have recently been associated both *in vitro* and *in vivo* with an increased Aβ42∶Aβ40 ratio [58]. The decreased levels of the other components were even more pronounced when we compared the amount of components incorporated into an active complex, as determined by the ability of a biotinylated γ-secretase inhibitor to capture the components. In general, the proportion of the components that were captured by this inhibitor was lower in adult brain membranes and the slight increase in PS2-CTF levels in adult brain was not reflected in increased amount of PS2-CTF in active complexes. One explanation for this could be that γ-secretase in lipid rafts may be less accessible to GCB and that γ-secretase localizes to lipid rafts in a higher proportion in adult than in embryonic brain [42].

Since age is a risk factor for Alzheimer's disease we also investigated whether γ-secretase activity as well as component levels changed with increased age of the adult rat. However, we found no differences in γ-secretase dependent production of AICD, NICD and Aβ40 and levels of γ-secretase components between young (2–3 month-old) and old (16–18 month-old) adult rat brain membranes. Again, we found no differences between non-incubated samples and samples incubated at 37°C in the presence of a γ-secretase inhibitor, indicating that there is no degradation of NICD or Aβ in our system. We cannot exclude the possibility that the production of Aβ42, which were below the levels of quantification, could be different between young and old rat brain.

Contrary to our study, Placanica *et al.* recently found decreased Notch processing and lower levels of γ-secretase components in aged mice [59]. The difference in Notch processing might be explained by the fact that they used a recombinant substrate whereas we studied the processing of endogenous Notch. In addition, we used 3 month-old rats as “young” animals, whereas they used 1 month-old mice and it is possible that the high γ-secretase activity and component levels that we detect in the embryonic rat brain to some extent remain at 1 month of age. Furthermore, the difference in species could also give different results. Placanica *et al.* also found a gender specific alteration in Aβ production at 24 months of age. In our young-old comparison we used a mixed population of male and female brains and we can therefore not compare our results with this study.

In conclusion we have demonstrated γ-secretase cleavage of five different substrates, APP, Notch1, N-cadherin, ephrinB and p75-NTR in embryonic rat brain membranes. The γ-secretase dependent ICD production was dramatically down-regulated in the adult and only cleavage of APP and Notch1 was detected. The ability to study γ-secretase cleavage of endogenous substrates in brain tissue provides an additional approach in which the effect and specificity of γ-secretase inhibitors and modulators could be examined.

## Materials and Methods

### Ethics Statement

All animals were handled in strict accordance with good animal practice as defined by the relevant national and local animal welfare guidelines, and all animal work was approved by the Animal Trial Committee of Southern Stockholm (S80-08, S63-07).

### γ-Secretase inhibitor, C99-FLAG and APP CTF-50

The γ-secretase inhibitor L-685,458 (Bachem) were dissolved in DMSO and added to the reactions (final concentration of 1 µM or 10 µM). The FLAG-tagged APP-based substrate C99-FLAG was treated with 70% formic acid in order to dissociate possible aggregates into monomers, dried in a vacuum centrifuge and resuspended in the sample (20 ng/sample). The APP CTF-50 (AICD) peptide (Calbiochem) was dissolved in MilliQ water to a concentration of 1 µg/µl and stored at −20°C until use when the peptide was further diluted in MilliQ water before subjection to SDS-PAGE.

### Antibodies

The following antibodies were used for immunoblotting: nicastrin, (MAB5556, Chemicon) raised against the final 18 C-terminal residues; PS1-CTF (MAB5232, Chemicon) recognizing the C-terminal loop region of PS1; PS1-NTF, (Calbiochem) raised against amino acids 1–65 of human PS1; UD1, raised against residues 1–11 of Pen-2 (a gift from Dr. Jan Näslund, Karolinska Institutet, Sweden); Aph-1aL (BioSite), raised against the C-terminal residues 245–265 of human Aph-1aL; C1/6.1, raised against the C-terminus of APP (a gift from Dr. Paul M. Mathews, Nathan Kline Institute, NY, USA); Ephrin-B1 (C-18, Santa Cruz Biotechnology) raised against the C-terminus of human ephrin-B1 but cross-reacts with ephrin-B2; N-Cadherin, (BD Biosciences) raised against residues 802–819 of mouse N-Cadherin; p75-NTR (Upstate) raised against residues 274–425 of rat p75-NTR; Cleaved Notch1 (Val 1744, Cell Signaling) recognizing the intracellular C-terminal domain of Notch1 only when cleaved between Gly1743 and Val1744 (γ-secretase cleavage); Notch1 (C-20, Santa Cruz Biotechnology) raised against the C-terminus of Notch1.

### Preparation of membrane fractions from rat brain

Male (3 month-old) and pregnant female Sprague-Dawley rats were obtained from B&K Universal (Sollentuna, Sweden) or Taconic (Hudson, NY). The rats were sacrificed by carbon dioxide and decapitated. For the young vs old comparison, rat brains from 2–3 month-old (young adults) and 16–18 month-old (old adults) Sprague-Dawley rats were obtained from Rockland Immunochemicals Inc. (Gilbertsville, PA). The rat brains were homogenized by 25 strokes at 1500 rpm using a mechanical pestle-homogenizer (IKALaborteknik RW20) in buffer containing 10 mM KCl and 10 mM Mops, pH 7.0 [29] supplemented with Complete™ protease inhibitor cocktail (PI) (Roche, Basel, Switzerland). The brain homogenates were centrifuged at 1000×g for 10 min and the post-nuclear supernatant was centrifuged at 100 000×g for 1 h. The membrane fractions were resuspended in 10 mM KCl and 10 mM Mops, pH 7.0, supplemented with 20% glycerol, aliquoted and flash-frozen in liquid N_2_. All centrifugation steps were carried out at 4°C.

### Cell lines and membrane preparation

Blastocyst-derived embryonic stem cells lacking PS1 and PS2 (BD8 cells) or one allele of PS1 and both alleles of PS2 (BD3 cells) [60] were cultured in Dulbecco's modified Eagle's medium (DMEM) supplemented with 10% fetal bovine serum, 1 mM sodium pyruvate, 0.1 mM β-mercaptoethanol and non-essential amino acids (Invitrogen). Cells were washed, harvested in PBS and collected by centrifugation. Subsequently, cells were subjected to membrane preparation as described above for rat brain.

### γ-Secretase activity assay

The membrane fractions were resuspended in buffer H (20 mM Hepes pH 7.0, 150 mM NaCl, 5 mM EDTA and PI). The protein concentration was measured by BCA protein assay kit (Pierce) and was kept at 3–4 µg/µl when examining ICD production and 1.25 µg/µl for Aβ40 measurements. The samples were incubated in the absence or presence of the γ-secretase inhibitor L-685,458 at 37°C for 8 or 16 h. In the cases where C99-FLAG was added, the incubations were performed in buffer H containing 0.4% CHAPSO. After incubation the samples were, in the case needed, concentrated using a vacuum centrifuge and analyzed by SDS-PAGE for the different ICDs.

### SDS-PAGE and western blotting

Equal amounts of protein (40–120 µg for ICD detection and 15–30 µg for detection of γ-secretase components) were mixed with 2× Laemmli sample buffer and either heated at 95°C for 5 min (for ICD production) or kept at room temperature for 20 min (for γ-secretase components) and subsequently separated by SDS-PAGE on 4–12% Bis-Tris or 16% Tricine gels. Proteins were transferred to nitrocellulose or PVDF membranes and probed with primary antibodies. Horseradish peroxidase-coupled secondary antibodies (Amersham) and ECL detection (Pierce and Millipore) were used for visualization. To verify that the amount of protein loaded onto the gel and transferred to membrane was similar in embryonic and adult membrane preparations Ponceau S staining was used. For quantification of γ-secretase components, the same membrane fraction at three different dilutions was loaded on the same gel as four different embryonic and four different adult membrane fractions. The intensity of all the bands was measured and the diluted membrane fractions were used to create a standard curve that was used for quantification of the samples. For quantification of AICD and APP CTFs, 125 pg, 250 pg and 500 pg of synthetic AICD peptide (APP-CTF 50, Calbiochem) were loaded on the same gel as activity assay samples from four different embryonic and four different adult membrane fractions. The AICD and the APP CTFs bands were quantified using the two standard points bracketing the intensity of the sample (two-point calibration). All quantifications were performed using a CCD-camera (Fuji LAS-3000).

### ELISA

De novo generation of Aβ40 was analyzed by commercial sandwich ELISA (Wako chemicals, Osaka, Japan) according to the manufacturer's instructions. The membrane fractions were incubated in buffer H and PI, with or without 5 mM 1,10-phenanthroline and 10 µM thiorphan for 8 or 16 h at 37°C and the reaction was stopped by adding RIPA buffer (150 mM NaCl, 1.0% NP-40, 0.5% sodium deoxycholate, 0.1% SDS, 50 mM Tris-HCl, pH 8.0) and boiling for 5 min. Control samples (NRC  =  no reaction control) were not incubated. The samples were centrifuged at 10 000×g for 5 min at room temperature and the supernatants were dispensed into the wells (100 µg protein/well) coated with BNT77 antibody directed against amino acids 11–28 of Aβ and incubated at 4°C overnight. The bound Aβ40 was detected by TMB reaction using HRP-conjugated BA27 antibody directed against the C-terminus of Aβ. All the measurements were performed in duplicates or triplicates and Aβ40 levels were calculated from the rodent synthetic Aβ(1–40) standard curve.

### Affinity capture of the γ-secretase complex

Membranes prepared from embryonic and adult rat brain were resuspended in buffer H containing 0.5% CHAPSO and PI. Magnetic streptavidin beads (Invitrogen) were added and the samples were incubated overnight at 4°C to remove biotinylated proteins. The samples were incubated with 200 nM of an affinity probe based on L-685,458 coupled to biotin by a cleavable linker (GCB) [52] for 20 min at 37°C with gentle shaking. As a negative control 10 µM L-685,458 was added to the sample and incubated at 10 min at 37°C prior to incubation with GCB. Magnetic streptavidin beads were added and the samples were incubated during 2 h at 4°C. The beads were washed with buffer H containing 0.5% CHAPSO and bound proteins were eluted in Laemmli sample buffer at 37°C for 45 min and subjected to SDS-PAGE.

### Statistical analysis

The absolute values for Aβ40 and Aβ40 production in membrane preparations from embryo and adult rats, and adult and old rats, respectively, was analyzed using two-tailed student's t-test. A threshold value of 0.05 was chosen for significance in all statistical analyses.
